# Seasonal forecasting of green water components and crop yield of summer crops in Serbia and Austria

**DOI:** 10.1017/S0021859618000047

**Published:** 2018-02-14

**Authors:** B. Lalić, A. Firanj Sremac, J. Eitzinger, R. Stričević, S. Thaler, I. Maksimović, M. Daničić, D. Perišić, Lj. Dekić

**Affiliations:** 1 Faculty of Agriculture, University of Novi Sad, Dositej Obradovic Sq. 8, 21000 Novi Sad, Serbia; 2 Institute of Meteorology, University of Natural Resources and Life Sciences, Gregor Mendel Str. 33, A-1180 Vienna, Austria; 3 Faculty of Agriculture, University of Belgrade, Nemanjina 6, 11080, Serbia; 4 Faculty of Sciences, University of Novi Sad, Dositej Obradovic Sq. 4, 21000 Novi Sad, Serbia; 5 Republic Hydrometeorological Service of Serbia, Kneza Višeslava 66, 11000 Belgrade, Serbia

**Keywords:** Ensembles of crop model output estimates, green water, seasonal weather forecast, staple food crops, water footprint

## Abstract

A probabilistic crop forecast based on ensembles of crop model output estimates, presented here, offers an ensemble of possible realizations and probabilistic forecasts of green water components, crop yield and green water footprints (WFs) on seasonal scales for selected summer crops. The present paper presents results of an ongoing study related to the application of ensemble forecasting concepts in crop production. Seasonal forecasting of crop water use indicators (evapotranspiration (ET), water productivity, green WF) and yield of rainfed summer crops (maize, spring barley and sunflower), was performed using the AquaCrop model and ensemble weather forecast, provided by The European Centre for Medium-range Weather Forecast. The ensemble of estimates obtained was tested with observation-based simulations to assess the ability of seasonal weather forecasts to ensure that accuracy of the simulation results was the same as for those obtained using observed weather data. Best results are obtained for ensemble forecast for yield, ET, water productivity and green WF for sunflower in Novi Sad (Serbia) and maize in Groß-Enzersdorf (Austria) – average root mean square error (2006–2014) was <10% of observation-based values of selected variables. For variables yielding a probability distribution, capacity to reflect the distribution from which their outcomes will be drawn was tested using an Ignorance score. Average Ignorance score, for all locations, crops and variables varied from 1.49 (spring barley ET in Groß-Enzersdorf) to 3.35 (sunflower water productivity in Groß-Enzersdorf).

## Introduction

There is no doubt that water shortages will contribute to the future global crisis of available resources. For the scientific community, permanent drought risk in production regions and freshwater shortages impose an ultimate goal to provide data and tools for better assessment and management of water resources (WWAP 2015). An important step towards realizing this goal is the introduction of a water footprint (WF) concept (Hoekstra *et al.* 2011). The water footprint is defined as the quantity of water used to produce a product or a service. In particular, the WF of any agricultural product is very much determined by plant yield and the volume of water used during the crop-growing period, which has three components: green water (precipitation, evapotranspiration (ET)), blue water (irrigation water transpired, return flow from drainage or runoff) and grey water (water required to dilute pollutants and to restore the quality standards of the water body). According to Ercin & Hoekstra (2012), climate change expected by 2050 will affect global agricultural production patterns and related WF of production and consumption but with highly different effects over regions. The global WF is expected to increase relative to the year 2000 according to all scenarios. In Europe, the most profound difference can be found between Eastern and South-Eastern Europe (ESEE) and Western Europe (WE). According to the scenarios, WF changes of consumption per capita relative to 2000 in ESEE will follow the trends expected in South Asia, Arab countries and most of Africa, the most vulnerable regions. Romania, Bulgaria, Serbia and Montenegro were net virtual water exporters in 2000 and it is expected that they will remain so in 2050. Gobin *et al*. (2017) determined that the WF for wheat can be up to five times larger for southern Europe compared with the high-yielding north-western European regions. Therefore, seasonal forecasting of WF and crop development could open up opportunities to establish mitigation measures to reduce the WF, particularly in the most vulnerable regions (Zoumides *et al.* 2014; Miguel-Ayala *et al.* 2016; Zhuo *et al.* 2016). An example of dynamic forecasting of WFs based on the Markov chain with a 1-year time step until 2030 can be found in Feng *et al*. (2016).

The climate of agricultural production regions in Serbia in the last decades of the 20th century is characterized by high variability including periods dominated by conditions that are too wet, and to a lesser extent too dry during crop-growing seasons. However, climate change during recent decades has altered the annual hydrological cycle, with prolonged droughts interspersed with extreme precipitation events and heat waves (Stričević *et al.* 2011*b*; Stričević & Djurović 2013; Gocic & Trajkovic 2014; Mihailović *et al.* 2015). Increased inter-annual variability in crop production imposes the need to implement efficient techniques such as better seasonal forecasting, as prescribed here, to adapt crop management for forthcoming weather conditions. A similar situation and changes are obvious for Austrian crop-growing regions, though improved growing conditions for crops with higher optimum growing temperatures (grain maize, soybean, sunflower, sugar beet) can be observed in cooler and more humid production regions in the vicinity of the Alps due to warming. During recent decades, the intensity of drought and heat waves has increased significantly, with lowland regions in the east and south being especially negatively affected with respect to crop yield, expressed also in a higher inter-annual variation of crop yield (APCC 2014).

As WF and crop development are the result of the interaction of two non-linear dynamic systems, atmosphere and plant, it is not possible to predict the exact status of either the atmosphere or the plant on monthly or seasonal timescales. To assess the uncertainties of long-term crop forecasting, non-linear aspects of crop modelling and the probabilistic character of forecasting should be taken into account (Schlenker & Roberts 2006; Higgins 2015; Higgins *et al*. 2016). This can be achieved through the use of an ensemble of crop models with the same initial conditions (related to weather, soil and plants) (Higgins 2015; Higgins *et al.* 2016), multiple runs of the same crop model with perturbed initial conditions or a combination of these strategies.

Results presented in the present paper are the outcome of a study whose main goal was to test the efficacy of seasonal weather forecasts (SWFs) in crop production. The objectives of the present study, elaborated in more detail in Lalic *et al.* (2017), can be summarized as follows: (1) to perform seasonal crop forecasting using deterministic and ensemble weather forecast as the input weather data for a crop model; (2) to assess the ensemble forecast's ability to provide a narrow range of feasible crop model outputs (CMO) and the associated green water footprint (GWF) of the crops (Mekonnen & Hoekstra 2010; Gobin *et al.* 2017) based on the ensemble spread (Toth *et al.* 2003); (3) to test seasonal CMO and GWF forecasting by comparing the deterministic and ensemble estimates with the results obtained using observed weather data; and (4) to analyse the CMO and GWF ensemble estimates distributions and evaluate them using Ignorance scores (Good 1952; Roulston & Smith 2002). More details about the methodology used for implementing and verifying ensemble crop forecasting, as well as results obtained for winter wheat seasonal forecasting in Serbia and Austria, can be found in Lalic *et al*. (2017).

The present paper presents further study results related to ensemble forecasting of green water components (GW), GWF and crop yield (in further text: ensemble GW and yield modelling) of selected important summer crops in Austria and Serbia – grain maize, sunflower and spring barley. The ensemble of crop model estimates is performed by using ensemble weather forecasts as the input weather data (perturbed initial/weather conditions) for multiple runs of one crop model.

In the current case study, the AquaCrop model (Raes *et al.* 2009; Steduto *et al.* 2009; Vanuytrecht *et al.* 2014) and ensemble weather forecast, provided by the European Centre for Medium-range Weather Forecast (ECMWF) were used for calculating an ensemble of estimates of GW and yield for summer crops at two selected locations of different crop-growing conditions in Austria and Serbia.

## Materials and methods

### Study area

Two locations were selected according to differences in WF scenarios among ESEE (Serbia) and WE (Austria) countries. Selected locations corresponded to the most important crop production areas: Groß-Enzersdorf (48°12′N, 16°33′E, 148 m a.s.l.) in Austria and Novi Sad (45°20′N, 19°50′E, 84 m a.s.l.) in Serbia, in which sunflower, maize and spring barley have been cultivated for many decades. Both locations are situated on the flat terrain of the southern and northwest Pannonia. However, weather conditions in Groß-Enzersdorf are strongly influenced by the presence of Alpine mountains in the west and southwest. Typical climates of the two study areas are continental or moderate continental, with mean annual temperatures of 11.5 °C in Novi Sad and 10.8 °C in Groß-Enzersdorf and mean annual precipitation of 647 mm in Novi Sad and 550 mm in Groß-Enzersdorf (reference period 1981–2010). There is high variability in temperature and precipitation, particularly during the spring, often expressed by excessive precipitation and increased frequency and intensity of hot and dry periods (Müller 1993; Lalic *et al.* 2012; APCC 2014).

### Meteorological data

For the purposes of the present study, the meteorological variables most commonly used in crop modelling were considered: daily maximum (*T*_max_) and minimum (*T*_min_) air temperature in °C, daily averaged relative air humidity (*r*, %), daily average incoming global radiation (*G*, J/m^2^), wind speed (*v*, m/s) and 24-h accumulated precipitation (*H*, mm). As the March–October period is the most important part of the growing season for summer crops, the selected time series (2006–2014) include data from 1 March to 1 October.

Historical records of these meteorological data for Novi Sad and Groß-Enzersdorf weather stations were obtained from the national weather service (Hydrometeorological Service of Republic of Serbia and Central Institute for Meteorology and Geodynamics of Austria, ZAMG). Owing to a lack of global radiation data, this variable was calculated using Prescot's empirical formula (Trnka *et al.* 2006).

Ensemble SWFs were provided by the ECMWF. The deterministic element of the Ensemble Prediction System is the deterministic – control forecast, i.e. control run (CR). A CR is a forecast model run without any perturbations of the initial conditions to analysis. Providing the initial conditions for the control analysis consists of collecting observations and interpolating data from irregularly spaced locations to the model grid and its objective analysis. Control run simulations were introduced to compare the results of applying ensemble weather forecasts for ensemble GW and yield forecasting with results obtained using deterministic weather forecasts.

The present study used ECMWF SWF products starting on 1 March for all available years and ensemble members (EMs) in the Meteorological Archival and Retrieval System (MARS). The seasonal forecast system of ECMWF began with 10 EMs in 2006 for 6 months and progressed to 50 EMs in 2014 for a 7-month forecast. The 24-h averaged values for selected meteorological variables from the start to the end of the forecast period were used for selected locations. The resolution of the seasonal ensemble forecast data was 0.5° × 0.5°. From those fields, geographically averaged values were extracted from the four nearest numerical points. Because of the specific terrain and some mountains in the vicinity of Groß-Enzersdorf, the selection did not match well with the observations. A comparison of the real topography with the static field of model orography, in a given horizontal resolution, helped with selection of the best option, which was one of the nearest numerical points in the southeast.

To implement ensemble forecasting of GW components (precipitation during the vegetation period and ET), GWF and yield and to compare it with its deterministic counterparts, two sets of meteorological input data based on the EMs) and CR were designed for the entire period of interest during the selected 9-year period. The results of ensemble GW and yield modelling were averaged over all EMs to obtain ensemble averages (EA) (Anderson *et al.* 2007). The results obtained using observed and CR datasets are designated OB and CR, respectively.

### Soil data

The main soil chemical and physical characteristics of top soil for the selected locations can be found in Stričević *et al*. (2011*a*) and Eitzinger *et al*. (2013), while hydrological characteristics are presented in Table 1. The soil type used for the simulation site in Austria (Chernozem) belongs to the dominating soil type over the Marchfeld crop production region where Groß-Enzersdorf is located. Its top horizon was formed by loess, with good water and nutrient storage capacity. However, below 1 m soil depth, sand and gravel occur and the groundwater table is located more than 3 m below the surface in most of the area, allowing no groundwater impact to the root zone of crops. The same type of soil, with similar chemical and physical properties, is present in the Novi Sad region. The only difference relates to a very deep top soil horizon and sub-horizon, having a favourable clayey soil texture, that may retain significant amounts of water used in the summer period by a deep, well-developed root system of field crops. The groundwater table is below 2 m in winter and much deeper in summer.

**Table 1.** Soil hydrological properties for selected locations in Serbia and Austria

Location	Depth (m)	Soil type	Texture	Field capacity (%)	Wilting point (%)	Pore space (%)
Novi Sad	0–0.3 >0.3	Chernozem	Loam	33.8 35.8	16.3 20.2	54.9 48.8
Groß-Enzersdorf	0–1	Chernozem	Silt Loam	35.0	21.0	43.0

### Crop data

For each location, major summer crops: spring barley, maize and sunflower were selected for ensemble crop simulation. Crop calendar and critical growth parameters (Table 2) were set using experimental field data collected for each region and using results from previous crop model studies (Stričević *et al*. 2011*a*; Eitzinger *et al*. 2013; Mirosavljević *et al*. 2015). Spring barley, maize and sunflower are common crops in the Marchfeld region, where maize is irrigated owing to frequent summer droughts caused by heat waves and strong winds. In Serbia in the Novi Sad region, maize and sunflower are very important industrial and fodder crops, grown mainly in rainfed conditions since yield benefit from irrigation is irregular. During the period 2006–2014, irrigation would have increased the yield only of maize in 1 year during an extreme drought (2012). Therefore, in the present research, the irrigation option was excluded in both locations. In Serbia, the sowing calendar may vary by up to 1 month (early April – early May) due to mean air temperature and soil moisture. Typical sowing periods are shown in Table 2.

**Table 2.** Planting (P) dates and growing degree-days (GDD) for selected crops and locations in Serbia and Austria

Location	Spring barley	Maize	Sunflower
	P/GDD	P/GDD	P/GDD
Novi Sad	5 Mar/1239	6–22 Apr/1530	6–22 Apr/1200
Groß-Enzersdorf	5–22 Apr/989	30 Apr–11 May/1311	05–22 Apr/1222

### Crop model application

The AquaCrop model version 5.0 (Raes *et al.* 2016) was used in the present study to calculate intensity of soil surface evaporation and crop transpiration (sum of both fluxes is denoted as crop evapotranspiration *E*_ta_ (mm/day)), water productivity for yield (WPet) as yield produced per unit volume of evapotranspired water (kg/m^3^) and yield (t/ha).

AquaCrop simulates yield response to water availability and is particularly designed to address conditions where water is a key limiting factor in crop production. The model is designed to work with limited number of crop and soil input parameters but to provide useful information in order to: improve soil moisture control practices (under rainfed conditions) and water productivity, optimize irrigation scheduling, mitigate yield loses under high variability of precipitation over the growing season and quantify the impact of climate variability and change on cropping systems.

AquaCrop was initially calibrated and validated against process-oriented crop models for the Marchfeld plain in Northeast Austria (Thaler *et al*. 2012; Eitzinger *et al*. 2013) but further calibration would be needed for a specific crop, irrigation management options and cultivar effects (Thaler *et al*. 2017). For the agroecological conditions of the Novi Sad region, AquaCrop was calibrated and validated against observed maize and sunflower data (Stričević *et al*. 2011*a*). More details of the parameterization of water balance and crop development processes in the AquaCrop model can be found in Steduto *et al*. (2009) and Raes *et al*. (2009).

For the AquaCrop simulations, standard crop management was assumed, including optimum fertilization. No other limiting factors such as pests and diseases or abiotic damages were considered in the simulation, except water and heat stress effects on crops. The crop model was run using SWFs (EM and CR datasets) and observed weather data (OB dataset) for Novi Sad (Serbia) and Groß-Enzersdorf (Austria) to obtain the ensemble of calculated GW and yields for maize, spring barley and sunflower.

Green water footprints (GWFs) of the selected summer crops for rainfed conditions were calculated according to Mekonnen & Hoekstra (2010):
1
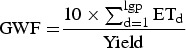

where lgp is the length of growing period (days) and ET_d_ (mm) is daily amount of ET.

### Verification statistics for ensemble green water and yield forecasting

The verification methodology, based on root mean square error (RMSE) and ensemble spread (a measure of deviation of each ensemble member from the ensemble mean) (SPRD) calculation (Toth *et al.* 2003) was used to evaluate the ensemble-based GW and yield forecast during the period of interest according to the methodology described in Lalic *et al.* (2017). As each EM was equally probable, RMSE as a measure of forecast accuracy was calculated for each year, comparing values of GW and yield estimates calculated using EMs, *Y*_i_, and observation-based results, *Y*^OB^, as follows:
2
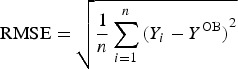

Deviation of the ensemble forecasts, *Y*_i_ from their mean, *Y*^EA^ is an important attribute of the ensemble-based calculations. Therefore, the SPRD for each year was calculated using the following formula:
3
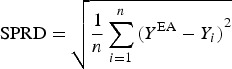

A comparison of Eqns (2) and (3) shows that an ideal ensemble forecast will have the same size of RMSE and SPRD, as for each EM the forecast value is equal to observed values. Accordingly, the simulation obtained using the ensemble forecast is more realistic if RMSE and SPRD values are similar.

The average values of GW and yield across all EMs, as well as the values obtained using the CR dataset, were calculated for each year and correlated with the corresponding values obtained using the OB dataset over the period 2006–2014. From the procedures described by Pielke (1984), the simulation could be considered more realistic if (a) RMSE obtained using simulated data (RMSE^CR^, RMSE^EA^) is less than the standard deviation of observed values (*σ*_OB_), and (b) standard deviation, *σ*, calculated using forecasted data (*σ*_CR_, *σ*_EA_) is close to that obtained using observed data, *σ*_OB_. In the present study, RMSE was calculated for both the EA and CR datasets for the chosen 9-year period, as it provides a good overview of a dataset with large errors weighted more than many small errors (Mahfouf 1990).

To assess and quantify GW and yield simulation uncertainties, the ensemble of estimates was transformed into a probabilistic forecast. For the variables which have normal (Gaussian) distribution, the success of the probabilistic ensemble forecast was analysed and evaluated by Ignorance score (Good 1952; Roulston & Smith 2002)
4
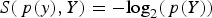

where *p*(*Y*) denotes the probability density of verification value of variable *Y*, which is the variable value calculated using the OB dataset. From Eqn (4) it follows that the lower the ignorance scores the better the simulation. Indeed, since the Gaussian distribution satisfies the 68–95–99.7 rule, the Ignorance score is <2.04 with probability 0.68, it is <4.21 with probability 0.98 and Ignorance score is >7.81 with probability 0.03. Hence, if ignorance is <2.04 the model is very good and if it is >7.81 model is not adequate.

Comparison of ignorance scores for different variables was enabled through the introduction of *Z*-scores (*Z* = (*Y* − *μ*)/*σ*; *μ* – ensemble mean), which implies that the probability density, *p*(*Z*) from Eqn (4) is the standard Gaussian density
5
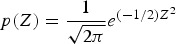

Knowledge of the probability distribution function offers deeper insight into the distribution of ensemble estimates. In general, if a normal distribution is an appropriate choice for yield distribution description, it implies that among all ensemble estimates one has the highest probability and this corresponds to the EA. A high-average Ignorance score and low standard deviation of ignorance, particularly over a long period of time, indicate that the chosen probability distribution function is not adequate. However, a high-average ignorance associated with high standard deviation can be a good indication of some effect which disrupts either the ensemble SWF or GW and crop model simulations, or both.

## Results

### Green water components

#### Precipitation

**Fig. 1.**
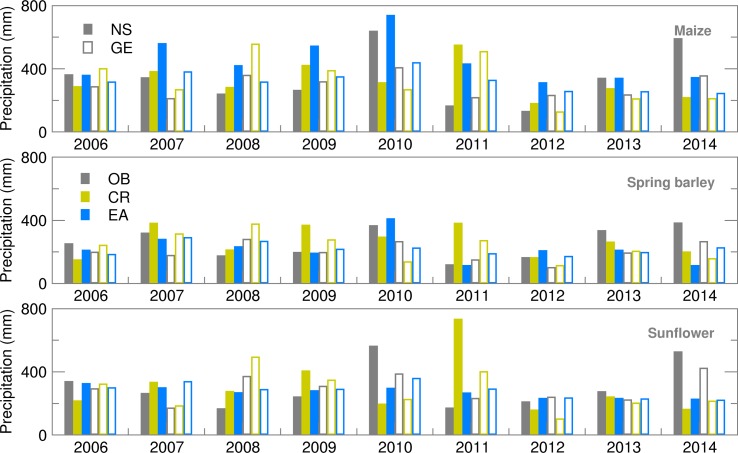
Precipitation during the growing season of maize, spring barley and sunflower obtained using observed (OB), control run (CR) and ensemble average (EA) datasets for 2006–2014 (Note: on all plots, filled bars correspond to Novi Sad (NS) and empty ones to Groß-Enzersdorf (GE); colours are distributed over datasets as follows: grey – OB, green – CR and blue – EA).

The highest deviation of precipitation from observations was identified for NS (in the Results and Discussion sections, due to frequent references to one or another location, the abbreviations NS and GE will be used for Novi Sad and Groß-Enzersdorf, respectively) in 2010, 2011 and 2014 (Fig. 1). According to 1981–2010 climatology, the average precipitation for NS was 647 mm. In 2010, annual precipitation was 1041.9 mm, in 2011 384.6 mm, while in 2014 it was 816 mm. In 2010, the observed precipitation (553 mm) exceeded climatological values (279.7 mm) from May to August. Selected years showed an overestimation for CR as well as EA in comparison with OB data (Fig. 1). The extreme heavy rain in May 2014, which caused massive floods on many rivers in Serbia, brought about an underestimation of precipitation amount. It is important to emphasize that EA was closer than CR to observation when extreme events were common, i.e. during dry weather or extreme precipitation.

**Fig. 2.**
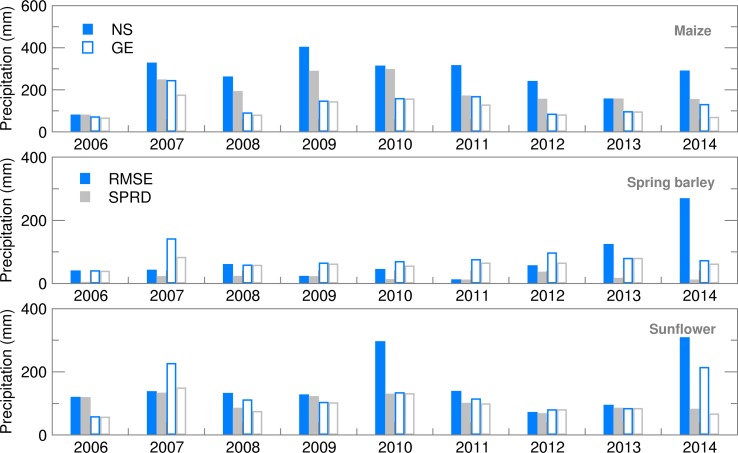
Root mean square error (RMSE) and ensemble spread (SPRD) for precipitation during the growing season of maize, spring barley and sunflower for 2006–2014.

In the absence of extreme weather events, different verification statistics indicators for precipitation (Fig. 2) in the same year are the result of different timings and duration of the vegetation period of the three crops. Results indicated a much better ensemble forecast of precipitation for summer crops in GE. An exception was 2007, which belonged to a year with very low precipitation during the growing season. Precipitation forecasting was particularly difficult in NS during the maize vegetation period (typically from early or mid-April until the end of October), which was confirmed by a high RMSE and SPRD and its difference. During the usually shorter growing period of sunflower, much lower deviations from the observed precipitation were obtained, with almost equal values of RMSE and SPRD, indicating good quality of the ensemble SWF. The best results were obtained for spring barley, as its growing period finishes before or just at the beginning of the high summer season. More details about the SWF used in the present study, particularly its RMSE and SPRD, can be found in Lalic *et al*. (2017).

**Fig. 3.**
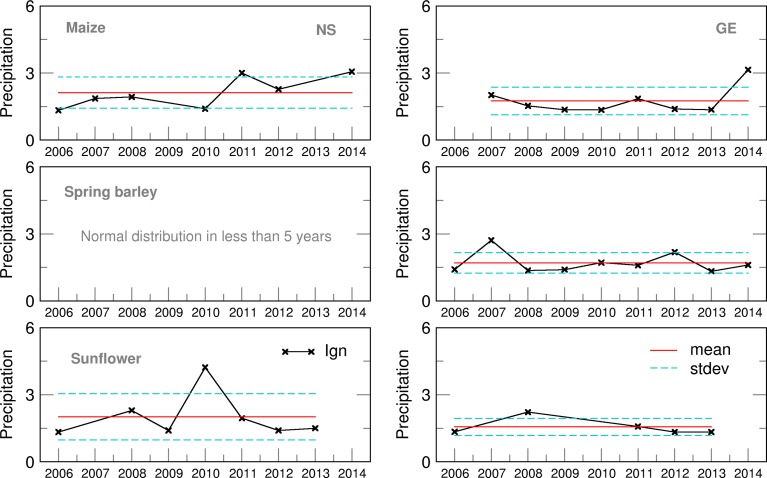
Ignorance score for Precipitation for maize, spring barley and sunflower for 2006–2014.

Probability density distribution of the ensemble estimates for both locations was, commonly, in accordance with the Gauss normal distribution except for spring barley in NS. Even if the year 2014 was not taken into account, average Ignorance score for GE was slightly lower than for NS (Fig. 3) with smaller standard deviations and variation over the years. This indicates lower uncertainties in precipitation forecast during the growing period of summer crops in GE. However, in both locations the highest ignorance was related to years/seasons with the amount of precipitation above or below the long-term average.

#### Evapotranspiration

**Fig. 4.**
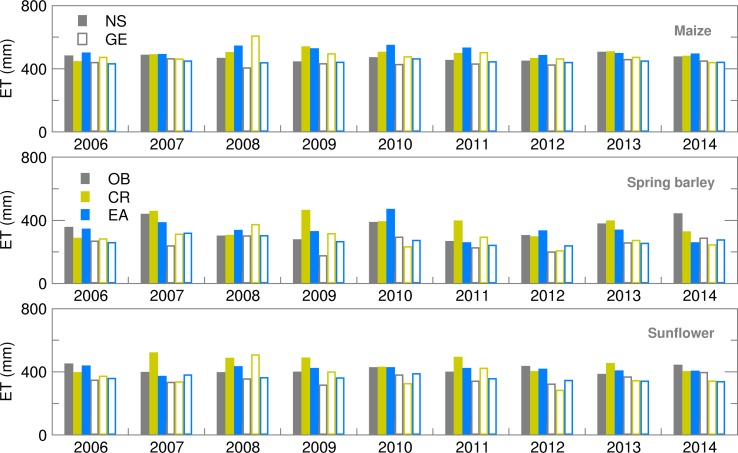
Evapotranspiration (ET) during the growing season of maize, spring barley and sunflower obtained using observed (OB), control run (CR) and ensemble average (EA) datasets for 2006–2014.

**Fig. 5.**
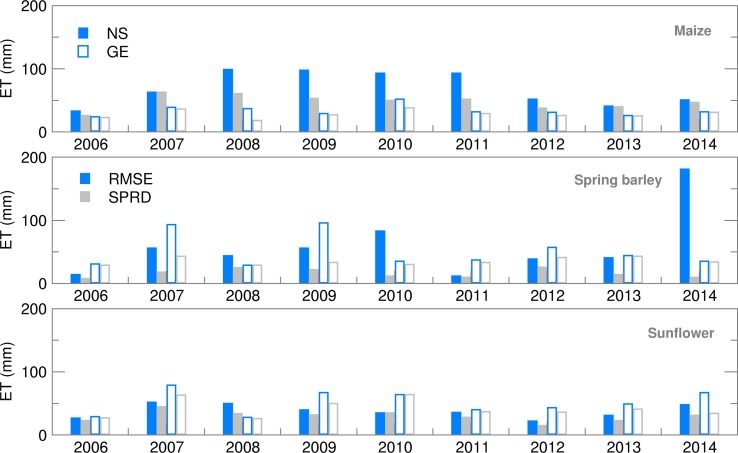
Root mean square error (RMSE) and ensemble spread (SPRD) for evapotranspiration (ET) during the growing season of maize, spring barley and sunflower for 2006–2014.

Accumulated ET is calculated as the sum of soil evaporation and canopy transpiration. Results obtained for maize and spring barley are in accordance with results obtained by Gobin *et al*. (2017) for 1992–2012. For both locations during the whole period of interest, high ET of maize was quite well forecast using both ensemble (EA) and deterministic (CR) forecasts (Fig. 4). Differences between RMSE and SPRD during 2008–2011 in NS (Fig. 5) indicate a high spread of ensemble estimates and deviation of EMs from OB-based calculations, i.e. high uncertainty of this forecast which was mostly the result of high precipitation spread during the maize-growing period. During the spring barley-growing season, ET simulations based on deterministic forecast (CR) commonly overestimated OB results.

Higher values but a similar pattern, as in the case of spring barley, were found in ET forecasting using the CR dataset during the sunflower-growing season. Ensemble estimates of ET for sunflower, in both locations, were more in accordance with OB-based simulations than in the case of maize and spring barley, while results for NS were slightly better than for GE.

**Fig. 6.**
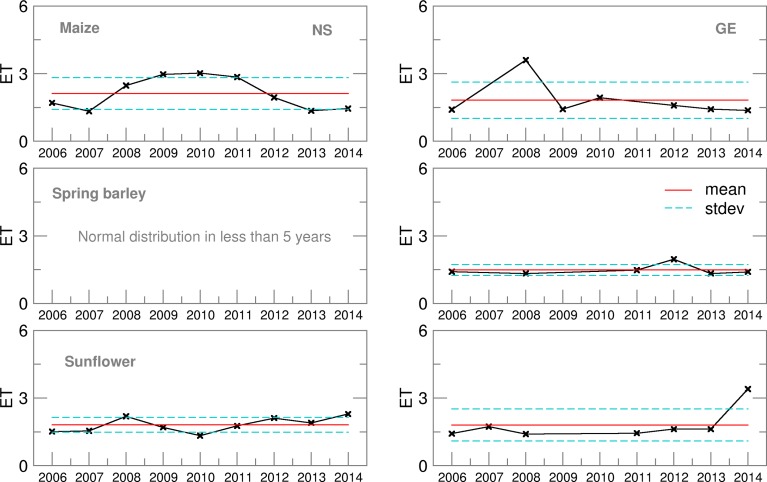
Ignorance score for evapotranspiration (ET) of maize, spring barley and sunflower for 2006–2014.

The Ignorance score for ET (Fig. 6) indicated a good performance of the ensemble forecast, particularly for sunflower and spring barley in GE. Similar averages, but higher inter-annual variation during the maize-growing season was the result of the high uncertainty of the ET forecast and its distribution deviation from the Gaussian function. A slightly lower performance of the probabilistic forecast was obtained for spring barley in NS, when the distribution could be considered normal only for 5 years (2006–2008, 2012, 2013), but with a high Ignorance score indicating low probability to obtain OB-based ET using a normal distribution of ensemble estimates.

#### Crop yield

**Fig. 7.**
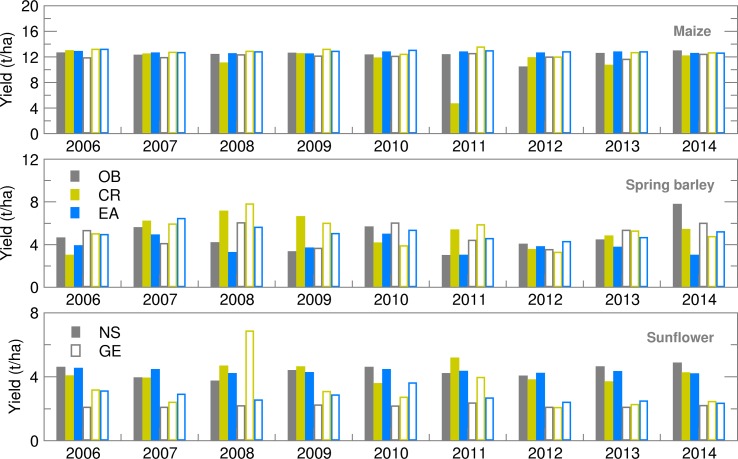
Yield calculated for maize, spring barley and sunflower obtained using observed (OB), control run (CR) and ensemble average (EA) datasets for 2006–2014.

**Fig. 8.**
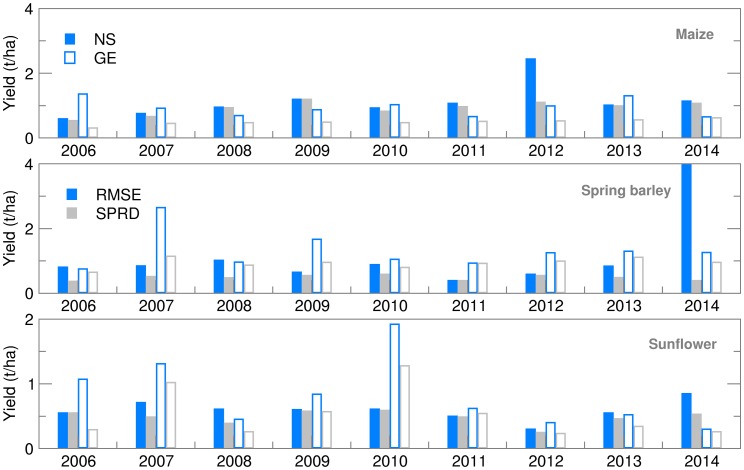
Root mean square error (RMSE) and ensemble spread (SPRD) for yield during the growing season of maize, spring barley and sunflower for 2006–2014.

Maize and spring barley yield calculated using the observed weather data for both locations were slightly (up to 15%) higher than those obtained by Gobin *et al*. (2017) using weather data for the 1992–2012 period. This is because calibrated crop parameters were used for the study area (Stričević *et al.* 2011*a*) and an appropriate sowing calendar that best suits the crop. Maize yield forecast using both ensemble and deterministic weather forecasts was very close to the OB-based yield (Fig. 7). The RMSE for maize was below 10% of yield, except in the extremely dry and hot growing season of 2012 in NS, when ensemble estimates for yield were greatly underestimated in comparison to OB-based results (Fig. 8). This could be the result of (i) weakness of the AquaCrop model to simulate yield under weather conditions that cause severe water stress (Heng *et al.* 2009; Nyakudya & Stroosnijder 2014) and/or (ii) greatly overestimated amounts of precipitation in the weather forecast (Fig. 1). Higher values of RMSE and SPRD, but lower differences among them for NS indicate better calibration of crop model for maize, while parameters ratio for GE indicates better performance of ensemble SWF. Spring barley deterministic forecast highly varies over the years and significantly differs from OB-based results, due to a higher estimate of precipitation in 8 out of 10 years. Root mean square error and SPRD and their difference for GE indicate the much higher deviation of ensemble estimates than for NS and lower forecast performance than for maize, almost for the same reason. Ensemble estimates for sunflower yield deviate less from OB-based results than CR forecasted yield, while deviations and SPRD obtained for NS are commonly lower than in GE.

**Fig. 9.**
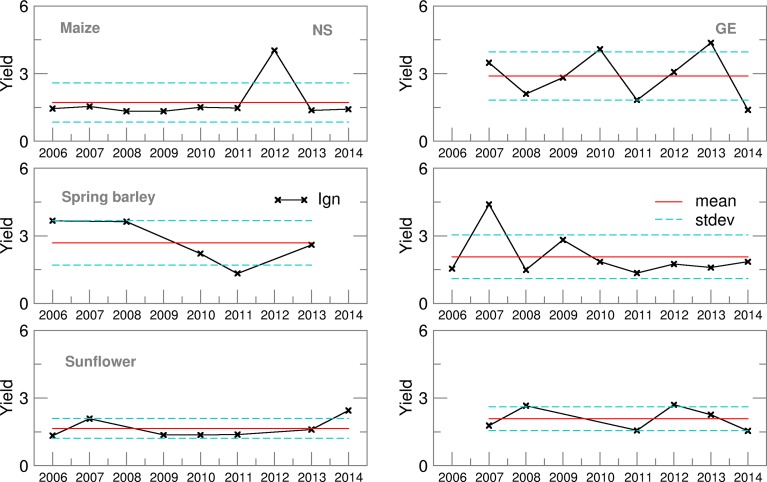
Yield Ignorance score for maize, spring barley and sunflower for 2006–2014.

Yield Ignorance score for all summer crops in NS is close to 2, with a standard deviation below 1 and low variability witnessing about the lower uncertainty of yield probabilistic forecast than in GE (Fig. 9). Average Ignorance score for GE is highly affected by 2006 yield forecast for maize and sunflower, which hardly fits Gauss distribution since for this year fewer EMs are available. Elimination of 2006 Ignorance brings GE scores into the NS ranges.

### Green water footprint and water productivity

**Fig. 10.**
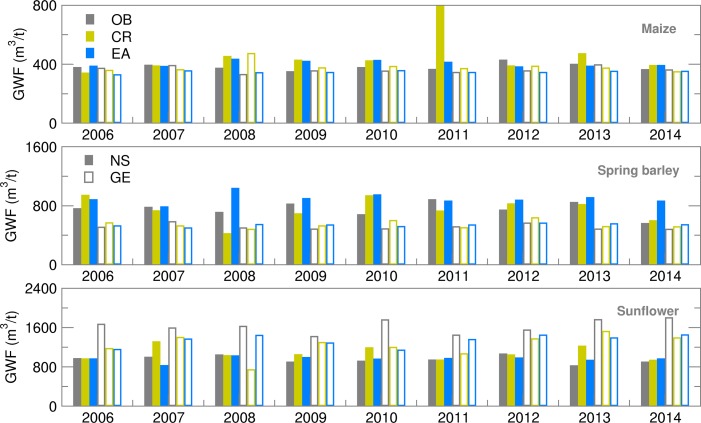
Green water footprint (GWF) for maize, spring barley and sunflower obtained using observed (OB), control run (CR) and ensemble average (EA) datasets for 2006–2014.

**Fig. 11.**
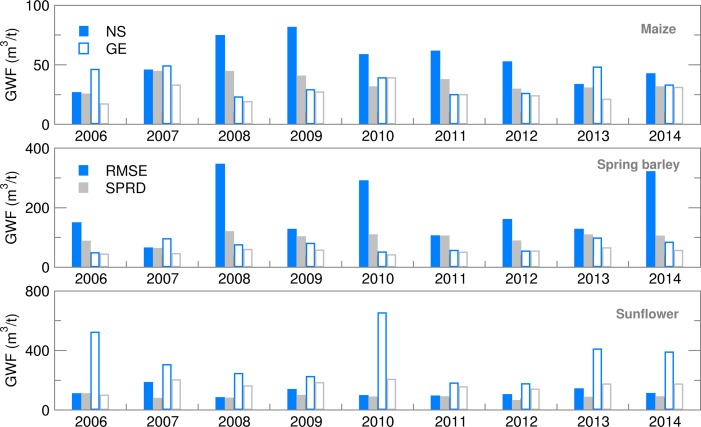
Root mean square error (RMSE) and ensemble spread (SPRD) for green water footprint (GWF) during the growing season of maize, spring barley and sunflower for 2006–2014.

The GWF calculated using Eqn (1) encompasses features of both ET and yield seasonal forecasting. The deterministic (CR) GWF forecast showed higher deviation from OB-based values for all summer crops and locations (Fig. 10). Green water footprint ensemble estimates for maize were frequently overestimated for NS but underestimated for GE. Average ensemble estimates of GWF produced an RMSE of up to 20% in comparison with OB-based values during the 2008–2012 period (Fig. 11). Deviation of GWF during the 2008–2011 period was due to greatly underestimated ET, while the high RMSE in 2012 was due to a significantly overestimated yield. The difference between RMSE and SPRD was high, particularly during the 2008–2012 period, because of high RMSE values. GWF for spring barley was 20–50% higher in NS than in GE due to generally lower yield levels in NS. While all forecast values (CR and EA) were close to OB values in GE, in NS, GWF ensemble estimates were commonly higher than OB values. Consequently, RMSE and differences between RMSE and SPRD for NS were two to three times higher than for GE. The RMSE/SPRD ratios were particularly high in 2008, 2010, 2012 and 2014 due to high ET ratios for the 2008–2014 period (excluding 2011), demonstrating that good ensemble forecasts of yield in 2009 and 2013 were enough to reduce the deviation of the calculated GWF. Sunflower GWF for GE was 30–50% greater than those calculated for NS due to lower general yield level in GE. Ensemble estimates were commonly underestimated, producing RMSEs which were approximately 25% of OB-based simulations. Deviations were particularly large in the following seasons in GE: in 2006, due to high variation of extreme temperatures and precipitation during July-August (July was extremely dry and hot, August extremely wet and cool); in 2010, due to air temperatures below average; in 2013–2014, due to very wet conditions with extreme summer precipitation.

**Fig. 12.**
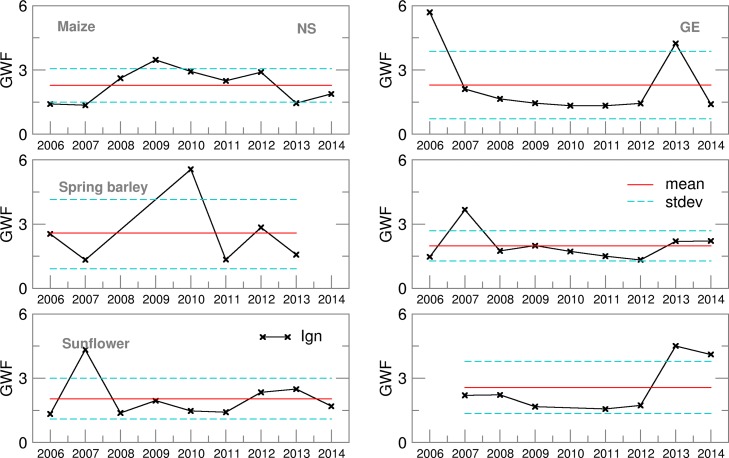
Ignorance score for green water footprint (GWF) for maize, spring barley and sunflower for 2006–2014.

The average Ignorance score of GWF for maize and sunflower in NS was 2 with a small standard deviation and low variability over the years (Fig. 12). A similar result was obtained in GE for spring barley and sunflower, excluding the results for sunflower in 2006 and 2010 (Table 3). A high Ignorance score and its significant variation for spring barley in NS was associated with years 2008, 2010 and 2014, in which the highest underestimations of simulated yield were obtained. Additionally, the ensemble forecast of precipitation during the spring barley-growing season did not give a normal distribution: the ET Ignorance score was two to three times larger than for other crops and locations. However, as the GWF probability distribution for those years can be considered as normal, and taking into account Eqn (1), it can be concluded that yield distribution and score had a more profound impact on GWF than precipitation and ET.

**Table 3.** Average Ignorance score, *S* and its standard deviation, *σ*^*S*^ for maize, spring barley and sunflower for the 2006–2014 period.

	Novi Sad						Groß-Enzersdorf					
	Maize		Spring barley		Sunflower		Maize		Spring barley		Sunflower	
	*S*	*σ*^*S*^	*S*	*σ*^*S*^	*S*	*σ*^*S*^	*S*	*σ*^*S*^	*S*	*σ*^*S*^	*S*	*σ*^*S*^
Precipitation	2.12	0.70	–	–	3.06 (2.02)^a^	3.10 (1.04)	1.75	0.61	1.70	0.46	2.63 (1.56)^b^	2.64 (0.39)
ET	2.12	0.71	–	–	1.81	0.33	1.82	0.81	1.49	0.24	1.80	0.72
Yield	1.72	0.87	2.69	0.99	1.65	0.44	4.00 (2.89)^c^	3.48 (1.07)	2.07	0.97	3.18 (2.08)^d^	2.94 (1.06)
GWF	2.28	0.78	3.38 (2.53)^e^	2.33 (1.62)	1.91	0.77	2.29	1.57	1.98	0.71	4.95 (2.57)^f^	5.56 (1.22)
WPet	2.19	0.74	5.45 (2.12)^g^	5.47 (0.86)	2.04	0.96	2.15	1.22	2.02	0.71	3.35	2.67

ET, evapotranspiration; GWF, green water footprint; WPet, water productivity for yield.

Values in brackets were obtained by excluding years with extreme scores

a 2014 (*S* = 10.34).

b 2014 (*S* = 7.96).

c 2006 (*S* = 12.89).

d 2006 (*S* = 9.75).

e 2008 (*S* = 6.41), 2014 (*S* = 7.15).

f 2006 (*S* = 18.76), 2010 (*S* = 7.77).

g 2008 (*S* = 11.84), 2010 (*S* = 8.82), 2014 (*S* = 16.16).

In the literature, water productivity for yield (WPet) is commonly expressed as the ratio of biomass and intensity of ET (Steduto *et al.* 2007). The linearity between crop biomass and water use (ET) is expressed in the form of many linear relationships (Hanks 1983). Similar results of linear regression analysis can be found for the relationship between final yield and ET (Allison *et al.* 1958; Hillel & Guron 1973). In AquCrop, crop yield is calculated as a product of harvest index and biomass (Foster *et al.* 2017), therefore WPet is inversely proportional to GWF with harvest index (which accounts for temperature and water stress) as a coefficient.

**Fig. 13.**
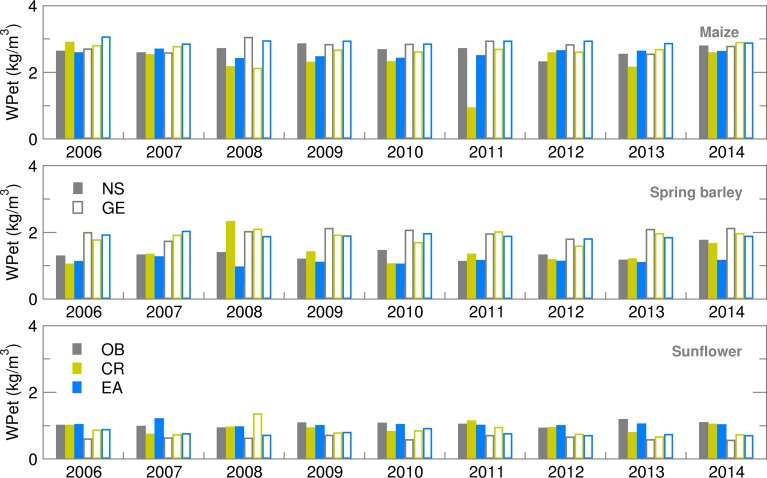
Water productivity (WPet) for maize, spring barley and sunflower obtained using observed (OB), control run (CR) and ensemble average (EA) datasets for 2006–2014.

**Fig. 14.**
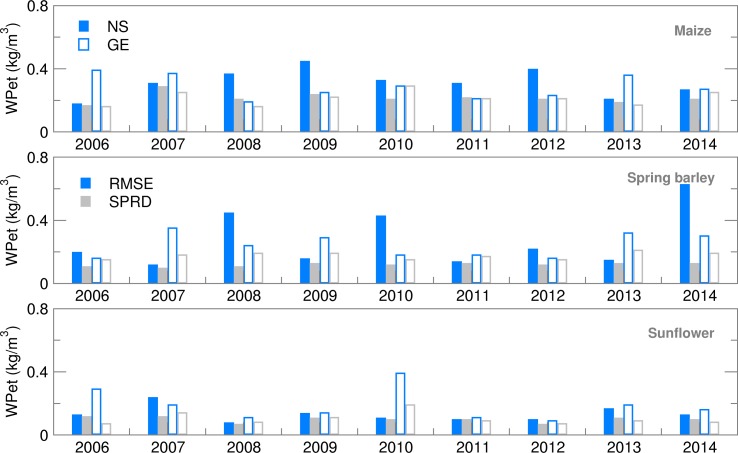
Root mean square error (RMSE) and ensemble spread (SPRD) for water productivity (WPet) during the growing season of maize, spring barley and sunflower for 2006–2014.

**Fig. 15.**
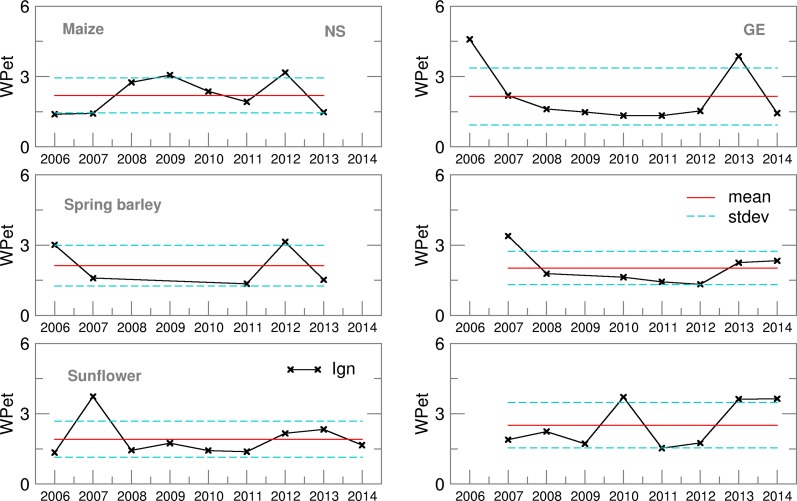
Ignorance score for water productivity (WPet) for maize, spring barley and sunflower for 2006–2014.

Water productivity for yield values, calculated using deterministic forecast (CR), deviated from OB-based values and varied over the years more than EAs (Fig. 13). This is the consequence of the setting for growing-degree-days (GDD) in the cropped file of the model, which calculates ET from sowing until the required GDD sum is fulfilled. For example, low temperatures in the model input files can prolong the modelled growing cycle significantly. The maize crop file for NS was calibrated for the most commonly sown hybrid with a long growing cycle. This is one of the reasons for a higher ET sum during the growing season of maize in NS, particularly using EA in comparison with GE. With the slightly higher yields at the Austrian location, this lower ET led to a higher calculated WPet for maize in GE than in NS, where ensemble estimates were often underestimated with respect to OB-based values. Similarly to maize, higher yield and lower ET during the spring barley-growing season in GE led to much higher WPet (and lower GWF) in GE than in NS for all the datasets used. The lowest calculated WPet were, in general, obtained for sunflower because it is an oil-producing crop, concentrating more energy in less mass, therefore producing lower yields in terms of weight than grain crops and giving a higher GWF and lower WPet (Figs 14 and 15). In comparison between the two sites, however, NS showed higher WPet values for sunflower, due to generally higher yield levels than in GE (Tables 4 and 5).

**Table 4.** Average values of selected variables, root mean squares error (RMSE), standard deviation, *σ* and coefficient of variation, cv, obtained using observed (OB), deterministic (CR) and ensemble (EA) for Novi Sad during 2006–2014 (*Note*: grey fields indicate results closest to OB-based results)

	OB	CR	EA	RMSE CR	RMSE EA	*σ* OB	*σ* CR	*σ* EA	cv OB	cv CR	cv EA
*Precipitation*											
Maize	345.0	326.6	453.6	219.6	192.3	164.6	106.2	131.4	47.7	32.5	29.0
Spring Barley	259.8	271.9	222.5	132.9	104.5	92.2	87.6	84.0	35.5	32.2	37.8
Sunflower	309.2	306.3	273.2	268.2	143.4	137.4	170.4	32.4	44.4	55.6	11.8
*Yield*											
Maize	12.4	11.2	12.5	2.7	0.8	0.7	2.4	0.1	5.6	21.2	1.1
Spring Barley	4.8	5.2	3.8	2.0	1.7	1.4	1.3	0.7	28.6	25.4	17.7
Sunflower	4.4	4.2	4.4	0.7	0.4	0.4	0.5	0.1	8.1	11.8	2.7
*Evapotranspiration*											
Maize	473.1	495.9	516.6	41.3	55.5	18.4	25.5	23.2	3.9	5.2	4.5
Spring Barley	352.7	371.9	342.4	88.3	74.2	62.8	64.0	59.8	17.8	17.2	17.5
Sunflower	417.0	455.0	418.6	75.1	24.8	22.7	44.1	18.7	5.5	9.7	4.5
*Water productivity*											
Maize	2.7	2.3	2.6	0.7	0.2	0.2	0.5	0.1	5.6	22.9	3.8
Spring Barley	1.4	1.4	1.1	0.4	0.3	0.2	0.4	0.1	13.4	26.5	6.8
Sunflower	1.0	1.0	1.05	0.2	0.1	0.1	0.1	0.1	7.4	12.7	6.0
*Green water footprint*											
Maize	384.0	484.8	406.4	233.9	43.0	21.9	204.1	19.2	5.7	42.1	4.7
Spring Barley	760.2	750.4	903.2	**161.6**	**187.2**	91.6	155.1	64.4	12.0	20.7	7.1
Sunflower	960.0	1085.9	963.1	199.3	85.7	72.7	126.6	51.6	7.6	11.6	5.3

**Table 5.** Average values of selected variables, root mean square error (RMSE), standard deviation, *σ* and coefficient of variation, cv, obtained using observed (OB), deterministic (CR) and ensemble (EA) for Groß-Enzersdorf during 2006–2014 (*Note*: grey fields defined as for Table 4)

	OB	CR	EA	RMSE CR	RMSE EA	*σ* OB	*σ* CR	*σ* EA	cv OB	cv CR	cv EA
*Precipitation*											
Maize	290.4	325.6	319.6	148.0	80.7	67.9	137.2	60.2	23.4	42.1	18.8
Spring Barley	202.1	231.9	217.6	94.1	50.9	55.6	82.1	37.3	27.5	35.4	17.1
Sunflower	293.0	275.8	282.3	122.7	94.8	80.8	116.1	45.2	27.6	42.1	16.0
*Yield*											
Maize	12.1	12.8	12.8	**0.8**	**0.8**	0.3	0.4	0.2	2.3	3.4	1.3
Spring Barley	4.9	5.3	5.1	1.5	1.0	1.0	1.2	0.6	19.8	23.5	11.9
Sunflower	2.2	3.2	2.8	1.7	0.7	0.1	1.4	0.4	4.0	43.3	13.7
*Evapotranspiration*											
Maize	436.1	487.2	443.8	78.2	19.0	17.1	45.7	8.2	3.9	9.4	1.8
Spring Barley	249.0	280.9	269.1	67.4	43.5	41.1	47.3	25.2	16.5	16.8	9.4
Sunflower	350.2	369.6	358.3	71.1	32.2	24.9	62.0	15.8	7.1	16.8	4.4
*Water productivity*											
Maize	2.8	2.6	2.9	0.4	0.2	0.2	0.2	0.1	5.4	7.8	2.2
Spring Barley	2.0	1.9	1.9	0.2	0.2	0.1	0.2	0.1	6.5	8.2	3.4
Sunflower	0.6	0.8	0.8	0.3	0.2	0.0	0.2	0.1	8.3	23.2	9.5
*Green water footprint*											
Maize	361.4	381.0	346.3	52.8	24.6	19.8	34.1	8.0	5.5	9.0	2.3
Spring Barley	509.9	539.9	535.6	56.8	50.6	35.4	46.8	18.5	7.0	8.7	3.4
Sunflower	1621.6	1237.3	1334.8	445.8	337.4	129.3	218.7	112.3	8.0	17.7	8.4

## Discussion

The results of the present study, using ensemble weather forecasting for simulating yield and water balance parameters of selected crops, are in accordance with regional statistics and results from other representative studies for that region (Todorovic *et al*. 2009; Araya *et al*. 2010; Gobin *et al*. 2017). Quality assessment of ensemble GW and yield forecast was made (a) comparing ensemble forecast estimates with results obtained using observed weather data (RMSE of the ensemble) and (b) measuring the width of ensemble spectra for the selected variable (ensemble SPRD).

Analysis and comparison of RMSE and SPRD with respect to the selected crop and variable of interest led to the following findings:

(a) Ensemble estimates of maize yield were, in general, better for GE than for NS. A possible reason for the lower forecasting for maize cultivation is the duration of its growing season and high probability of temperature and water stresses appearing in that period.
(b) Spring barley ensemble forecast statistics varied significantly among locations, seasons and variables. While results for precipitation and ET were very good (particularly for NS), ensemble estimates for yield, GWF and WPet significantly differed from OB-based results.
(c) Sunflower ensemble forecasts for all variables of interest produced the best or the second best result in comparison with maize and spring barley, particularly in NS.

Ensemble forecasting of GW and crop yield under frequently occurring extreme weather events is a particular problem and challenge, which is clearly seen at both locations, as the crop models often have difficulty simulating the impacts of extreme weather conditions on crop growth processes (Eitzinger *et al.* 2013; Lalic *et al.* 2014). Additionally, the forecast of extreme weather events is challenging *per se*. Hence, it is important to have a clear picture of the impacts of either SWF uncertainty or the crop model capacity to reproduce ensemble estimate-based impacts on crops.

Probability distribution analysis of ensemble estimates and Ignorance score of observation-based GW and yield simulations show consistent results. Average Ignorance score variation was lowest for spring barley ET in GE and highest for sunflower WPet at the same location. These results agree with the Ignorance score for maize yield (*S* = 1.36 and *σ* = 1.35) simulated by the CERES-Maize model (Higgins 2015). A high average ignorance associated with a high standard deviation in the case of spring barley in NS (for all variables except yield) and sunflower in GE (for all variables except ET) could be a good indicator of effects which, systematically, disrupt either ensemble SWF or simulation of selected variables, or both.

Comparison of deterministic and ensemble GW and yield forecast over the whole period of interest (2006–2014) indicates that deviation of ensemble estimates from observation-based simulations is smaller than in the case of deterministic ones. The standard deviation of ensemble estimates was low but usually closer to the standard deviation of observation-based results than results obtained using CR data. The variability of precipitation, yield and ET was of the same order of magnitude for all datasets, while for WPet and GWF much better results (lower variability and closer to OB results) were obtained for ensemble estimates.

Results obtained for ensemble forecasting of GW, GWF and yield indicate that use of ensemble forecast as input weather data in crop models is highly justified.

Seasonal forecast of GW and yield based on deterministic forecast (CR), even less demanding technically, produce commonly higher deviation from results obtained using observed weather data, with respect to ensemble estimates.

Transfer from an ensemble of estimates to the probability distribution of GW and yield simulations offers the possibility to identify the forecast variable for which the Ignorance score is the lowest. Use of historical data to calculate probability density for observed values or values based on meteorological observations gives additional information about ensemble forecast and its distribution for future use.

The present study has practical implications, especially for agricultural management and agricultural policy. First, many crop management options and timings depend on precipitation patterns and their forecasts, affecting soil wetness and soil workability. Optimizing machinery use, fertilizing and plant protection measures, harvest timing and others can have significant economic relevance for farmers as well as environmental effects. In case of irrigation agriculture, better precipitation forecast performance will improve the efficiency of irrigation water use and related economic aspects of irrigation. In that context, the effects on blue WF as well as irrigation water demand in relation to optimized irrigation schedules – based on improved seasonal forecasts – should be further investigated under the cropping regimes in the case study regions.

## Conclusions

The study shows that SWFs, although with some inert uncertainties, have the potential for applications in agricultural decision support, especially by its implementation in operational monitoring and warning systems. For example, at the policy level, it will help to improve regional seasonal yield forecasts, forecasts for upcoming crop damage risks (e.g. by drought and heat periods) or for irrigation water management in order to set early policy measures. At the farm level it can help for the more efficient planning of machinery use (e.g. during sowing and harvest times), for better irrigation scheduling, for soil and plant protection measures – all contributing to more sustainable and resource efficient farming practices.

Weaknesses related to extreme weather events can be overcome by using monthly and short-range NWP, during the integration period of SWF, but also by improving crop model capacity to simulate plant development as well as cropping risks caused by adverse weather conditions.
